# Supplementary figures and images for: Potential role of cellular miRNAs in coronavirus-host interplay
Source: PeerJ. 2020 Sep 14;8:e9994. doi: 10.7717/peerj.9994 (PMC7497610; doi:10.7717/peerj.9994)

**A**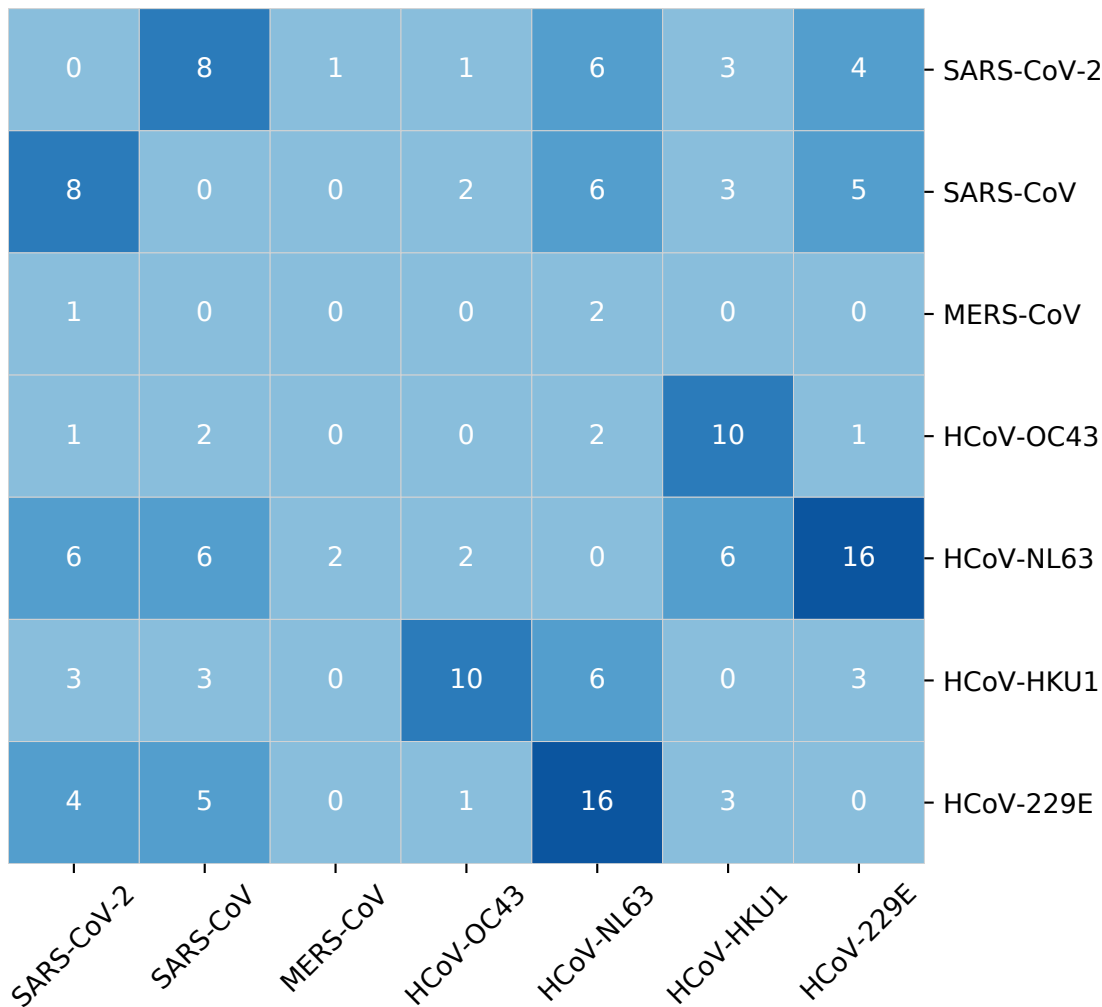**B**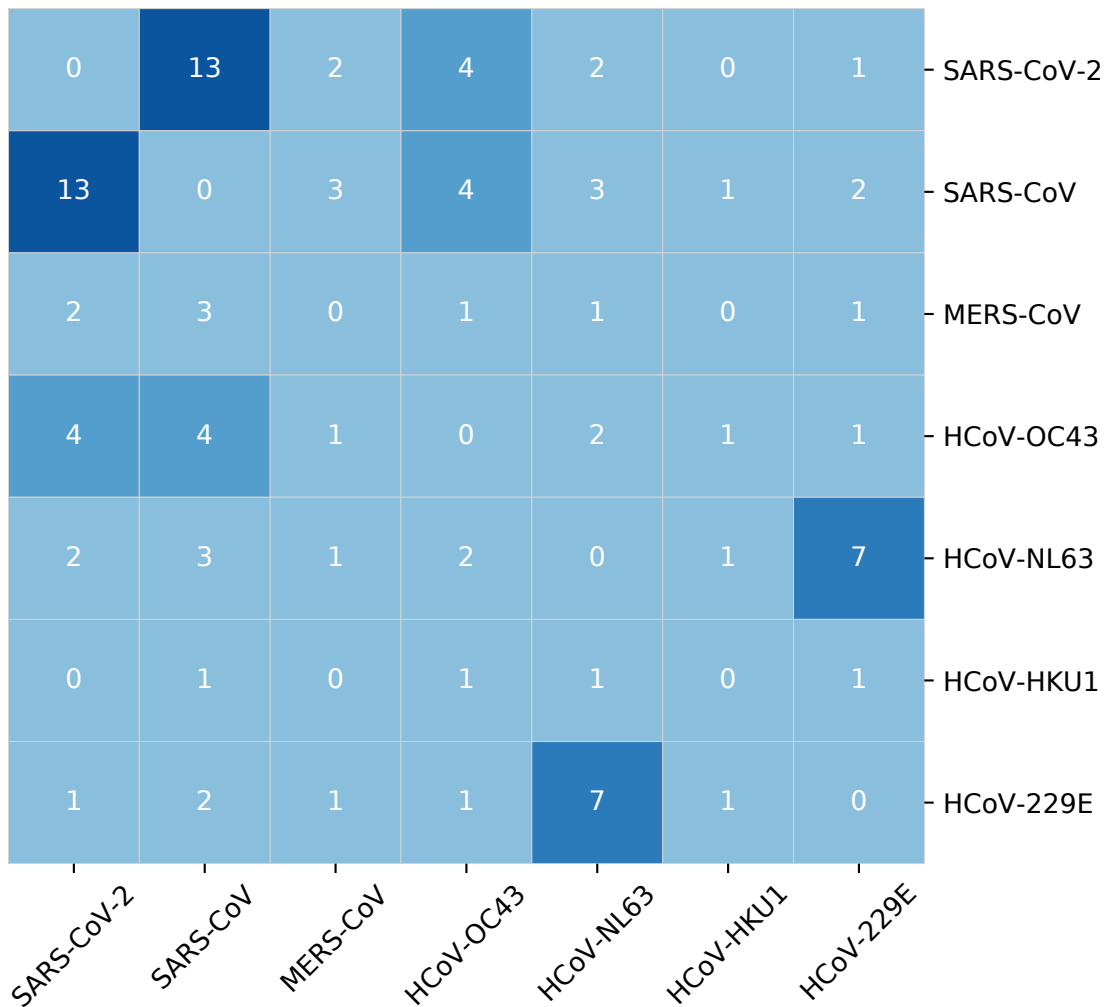**C**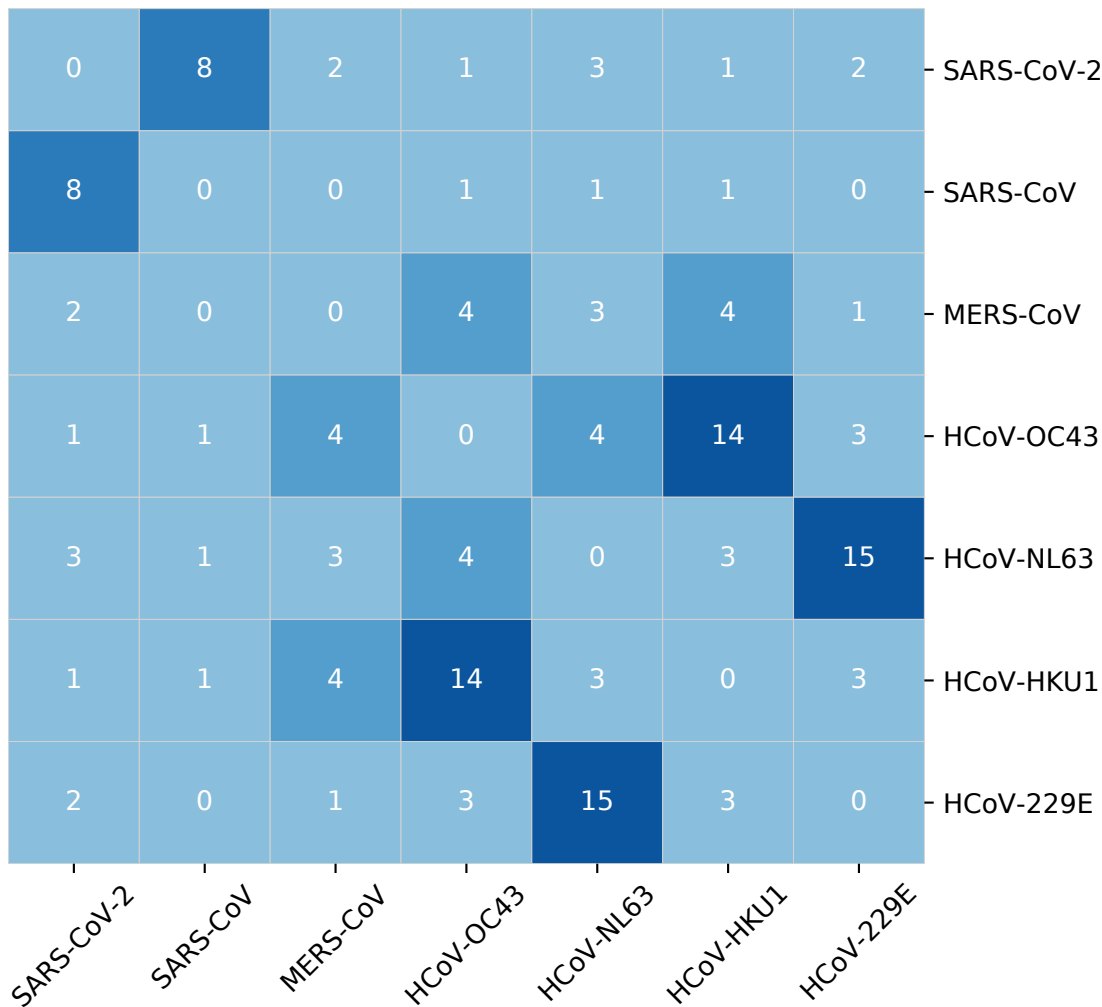**D**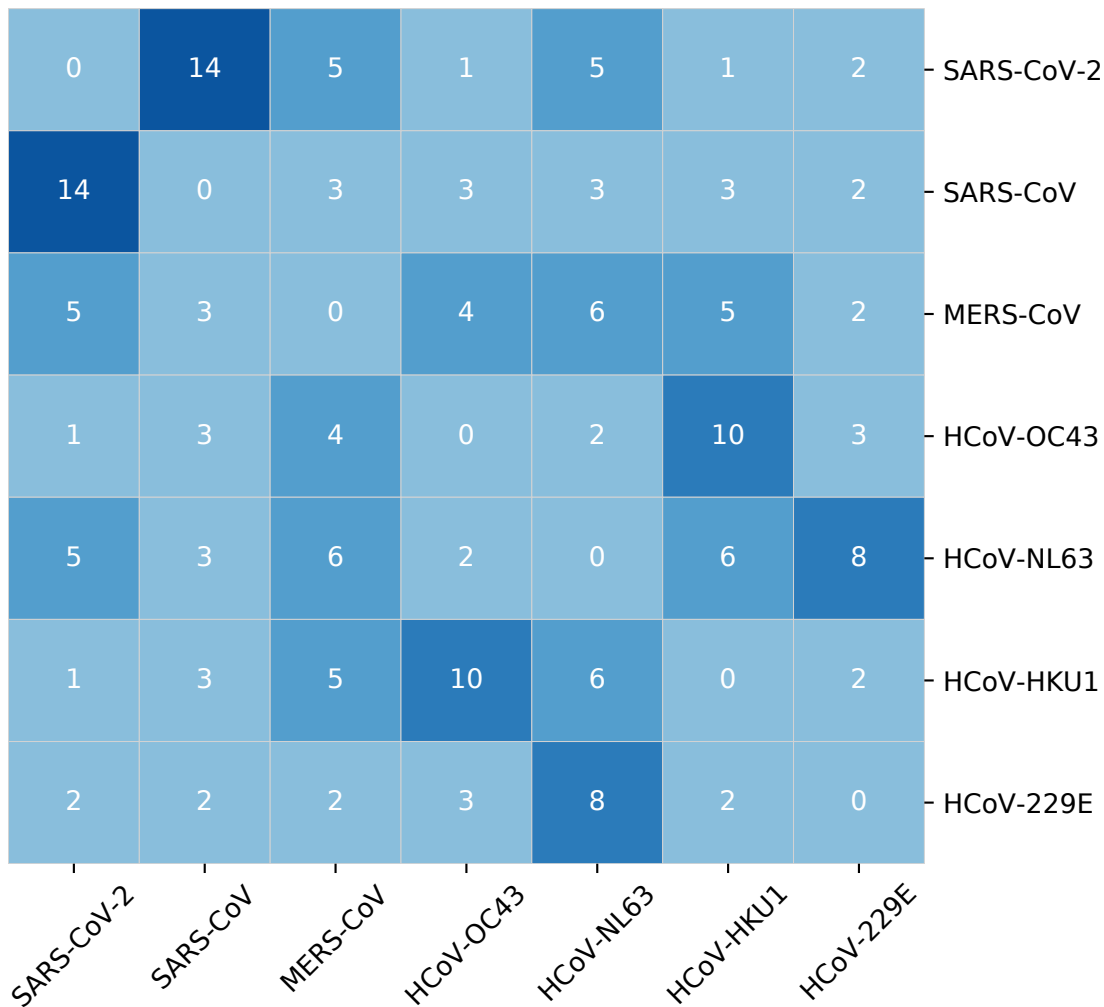

Supplement: Supplemental Information 1 — (A) hsa-miR-21-3p. (B) hsa-miR-16-5p/195-5p/424-5p. (C) hsa-miR-3065-5p. (D) hsa-miR-421. [file peerj-08-9994-s001.pdf]
